# Exploring the Potential Use of Wearable Devices as a Prognostic Tool among Patients in Hospice Care

**DOI:** 10.3390/medicina58121824

**Published:** 2022-12-12

**Authors:** Yaoru Huang, Muhammad Ashad Kabir, Umashankar Upadhyay, Eshita Dhar, Mohy Uddin, Shabbir Syed-Abdul

**Affiliations:** 1 Department of Radiation Oncology, Taipei Medical University Hospital, Taipei 110, Taiwan; 2 Graduate Institute of Biomedical Materials and Tissue Engineering, College of Biomedical Engineering, Taipei Medical University, Taipei 110, Taiwan; 3 School of Computing, Mathematics and Engineering, Charles Sturt University, Bathurst, NSW 2795, Australia; 4 Graduate Institute of Biomedical Informatics, College of Medical Science and Technology, Taipei Medical University, Taipei 106, Taiwan; 5 International Center for Health Information Technology, College of Medical Science and Technology, Taipei Medical University, Taipei 106, Taiwan; 6 Faculty of Applied Sciences and Biotechnology, Shoolini University of Biotechnology and Management Sciences, Solan 173212, Himachal Pradesh, India; 7 Research Quality Management Section, King Abdullah International Medical Research Center, King Saud bin Abdulaziz University for Health Sciences, Ministry of National Guard-Health Affairs, Riyadh 11481, Saudi Arabia; 8 School of Gerontology and Long-Term Care, College of Nursing, Taipei Medical University, Taipei 110, Taiwan

**Keywords:** wearable devices, prognosis, hospice care, palliative care, terminal care, colorectal cancer, non-small cell lung cancer

## Abstract

*Background:* Smartphones and wearable devices have become a part and parcel of the healthcare industry. The use of wearable technology has already proved its potentials in improving healthcare research, clinical work, and patient care. The real time data allows the care providers to monitor the patients’ symptoms remotely, prioritize the patients’ visits, assist in decision-making, and carry out advanced care planning. *Objectives:* The primary objective of our study was to investigate the potential use of wearable devices as a prognosis tool among patients in hospice care and palliative care, and the secondary objective was to examine the association between wearable devices and clinical data in the context of patient outcomes, such as discharge and deceased at various time intervals. *Methods:* We employed a prospective observational research approach to continuously monitor the hand movements of the selected 68 patients between December 2019 and June 2022 via an actigraphy device at hospice or palliative care ward of Taipei Medical University Hospital (TMUH) in Taiwan. *Results:* The results revealed that the patients with higher scores in the Karnofsky Performance Status (KPS), and Palliative Performance Scale (PPS) tended to live at discharge, while Palliative Prognostic Score (PaP) and Palliative prognostic Index (PPI) also shared the similar trend. In addition, the results also confirmed that all these evaluating tools only suggested rough rather than accurate and definite prediction. The outcomes (May be Discharge (MBD) or expired) were positively correlated with accumulated angle and spin values, i.e., the patients who survived had higher angle and spin values as compared to those who died/expired. *Conclusion:* The outcomes had higher correlation with angle value compared to spin and ACT. The correlation value increased within the first 48 h and then began to decline. We recommend rigorous prospective observational studies/randomized control trials with many participants for the investigations in the future.

## 1. Introduction

Palliative care is a specialized medical care that provides support in both acute-care hospitals and the community for patients with advanced diseases [1]. Life expectancy, as “how much time left”, is one of the critical questions among patients and their families, especially for the patients in palliative care [2]. The main goal of palliative care is to enhance patients’ quality of life at the end of their lives and lessen the grief felt by their family members so that patients can pass away peacefully and comfortably [1,3,4]. As cancer patients in palliative care are often with higher burden and have more demands in daily care, so not only patients and their families need more information in decision-making and life arrangements but also clinicians need to make a proper care plan based on the prognosis [2].

Performance Status (PS) is a score that is used for comprehensive evaluation of a patient’s level of function and daily activities. For the majority of clinical studies, PS is as important as an eligibility requirement, a comorbidity covariate, or a trial endpoint [5,6]. In the literature, several prognostic scores have been utilized in order to estimate survival of the patients in palliative care, including Palliative prognostic Index (PPI) [7,8], Palliative Prognostic Score (PaP) [8], Palliative Performance Scale (PPS) [9], and Karnofsky Performance Status (KPS) [10]. Without prognostic models, physicians can have the tendency to overestimate survival, which may have detrimental effects on the patients and their families [11]. However, these traditional oncology assessments have only yielded modest discrimination [6,10]. In addition to this, these scales are subjective, prone to bias, and have a high inter-observer variability [12].

Many nations have placed a high priority now on improving palliative care delivery, both in terms of access and quality [13]. In this regard, wearable technology can play an important role in improving healthcare accessibility, especially in palliative care [14]. Novel digital solutions, such as smartphones and wearables can be used to obtain objective patient-related parameters in a non-invasive, continuous, and real-time manner [15]. Wearable technology emerged as an innovative solution for healthcare issues in the recent years and now has become a part and parcel of the healthcare industry [16]. In the literature, numerous diseases and patient populations have been studied using wearable technology, including cardiovascular diseases [17], diabetes mellitus (DM) [18], chronic obstructive pulmonary disease (COPD) [19], Parkinson’s disease [20], cancer [21], and mental disorders [22]. Due to the real time data access and monitoring, wearable technology has tremendous applications in tertiary care hospitals/hospices for the care and treatment of critically and terminally ill patients and supporting the work of their care providers. This real time data captured through wearable devices and sensors allow the care providers to monitor the patients’ symptoms remotely, prioritize the patients’ visits, assist in decision-making, and to do advanced care planning [23]. These devices collect data from patients on a range of health-related issues, including blood sugar levels, exercise regime, sleep cycles, and emotional state [24]. By using sensors to record and measure biological variables, large digital datasets are created, which are suitable for real-time transmission to healthcare and non-healthcare experts. These applications are likely to have an impact on clinician–patient interactions and health care economic models [25]. Hence, wearable devices can be utilized for tracking and evaluating the symptoms in advanced diseases [26]. In addition, this wearable technology is quite helpful in providing relevant knowledge and education to the patients and their caregivers about hospices and end-of-life care, e.g., symptom management, side effects, and other issues. Moreover, by harnessing the power of Artificial Intelligence (AI) and big data analytics, the real time data collected through wearable devices/sensors could also be combined with the medical records to develop the risk prediction models [23].

Wearable devices can be used to objectively measure performance status in patients with advanced cancer [27]. Continuous health monitoring via wearables was found to be feasible in a recent study of palliative cancer patients with one-year prognosis [15]. According to our previous research in this domain, wristband actigraphy has the potential to predict end-of-life admission outcomes in cancer patients receiving palliative care [28]. Thus, there is an opportunity to investigate the potential impact of wearable devices among hospice/palliative care patients. The primary objective of our study was to investigate the potential use of wearable devices as a prognosis tool in hospice or palliative care patients, so we examined the utility of wearable devices in the continuous monitoring of palliative care patients from admission to their discharge in the hospice ward. As the secondary objective, we also examined the association between wearable devices and clinical data in the context of patient outcomes, such as discharge and deceased, at various time intervals.

## 2. Methods

### 2.1. Study Design and Setting

This was a prospective observational study conducted at the hospice/palliative care ward of Taipei Medical University Hospital (TMUH) in Taiwan. The main purpose of the study was to continuously monitor the hand movements of patients via actigraphy devices from admission to their discharge in the hospice ward. This trial study was approved by the Taipei Medical University-Joint Institutional Review Board (TMU-JIRB No. N201910041).

### 2.2. Recruitment

All patients were admitted to the hospice/palliative care ward between December 2019 and June 2022. The patients were screened for recruitment based on the following inclusion and exclusion criteria. Inclusion criteria included: (1) age over 20 years old, (2) major diagnosis as solid cancer and confirmation from two oncologists as terminal-staged, and (3) agreed on hospice care and Do-Not-Resuscitate (DNR). The exclusion criteria included: (1) dying symptoms or death estimate within 1 day at admission, (2) diagnosed with cancer of unknown origin, and (3) transfer to other wards or units after admission. A written consent for participation was required and signed by the patients or their next of kin, if the patients were unconscious and/or unable to express themselves clearly. The patients had the right to withdraw from the study at any point of time and the data would be erased accordingly.

### 2.3. Wrist Actigraphy

The selected the actigraphy device (model no. XB40ACT) was supplied by K&Y Lab (National Yang-Ming University, Taipei, Taiwan) and validated in a previous study [29]. The actigraphy device weighed 7 g with 4.4 × 1.9 × 0.8 cm dimensions. The device was used to collect three-dimensional data of gravitational acceleration, angular change, and spin change of a patient’s hand motion every second and transform the data into three different statistical parameters, namely *ACT* (activity level), *angle* and *spin*. The collected data were transferred to a data server once a week using Bluetooth, as the battery of the device lasted for 14 days only.

### 2.4. Data Collection and Acquisition

The patients recruited in this trial wore actigraphy devices on their wrists with silicon wristbands until they were discharged or deceased. The devices’ data were collected wirelessly (using Bluetooth connection) through its synchronized mobile application. The devices collected patients’ activities for 24 h a day and 7 days a week during the hospital admission. The patients’ data was retrieved with three variables: ACT, angle, spin and transferred to the server device. If any patient stayed longer than 10 days, a second wearable device (WD) was replaced on the 11th or 12th day of stay. Finally, when patients were discharged from the hospital or at the time when they were declared dead by physicians, the device was retrieved from them.

### 2.5. Clinical Information

The patients’ clinical information included date of admission, date of discharge, medications taken during the patients’ stay, length of stay, gender, age, comorbidities, whether any surgical procedures were performed, as well as diagnosis, patient ID, and device ID. To predict each patient’s chance of survival at the time of admission, performance scores were also captured using traditional prognostic tools, such as KPS, PPI, PaP, and PPS, but this study did not take performance scores into consideration.

### 2.6. Data Analysis

Actigraphy data were recorded as ACT, angle, and spin. The data was downloaded from the server and pre-processed. Descriptive statistics were applied for all the study variables to classify the data for better visualization. The correlation between the clinical information (Diagnosis, Admission cause, Use of Sedatives, Use of Opioids, and Use of Antipyretics) and survival outcome were also analyzed using Cramer’s V correlation.

We prepared four different datasets of different time intervals, i.e., 12 h, 24 h, 48 h and full dataset to classify the data of patients on the basis of collected wearable data. We examined the association of actigraphy data (i.e., ACT, angle, and spin) with the survival outcomes of the patients (i.e., discharged and expired). We tested whether there are any significant differences in total ACT, Spin, and Angle values for May be Discharge (MBD) and expired patients, in different time intervals using independent sample *t*-test. We prepared the pairplot using the python to visualize the relationship between the MBD and expired using the accumulated data of ACT, angle, and Spin.

## 3. Results

### 3.1. Demographics of Study Population

From 11 December 2019 to 30 June 2022, a total of 78 patients were recruited to TMUH’s hospice care unit, with 46 male and 32 female patients. The study was successfully completed by selected 68 patients. The remaining ten patients were removed due to missing or incomplete data and due to the failure to sync the device with the smartphone that resulted in data not being captured. The patients’ ages ranged from 39 to 92 years where the mean age was 72.1 years and 58.97% of the patients were males in the study. Colorectal cancer and non-small cell lung cancer were the top two cancers in terms of recruitment, accounting for 24.35% and 17.95% of all cancers respectively. Furthermore, aggravating cancer-related symptoms were the most common reason for admission (56.41% of the patients in this study), concomitant diseases were another common reason for admission, and no patient was admitted for a non-medical reason.

The concomitant diseases or symptoms were defined as less cancer-related but included infections from lungs or urinary tract, general weakness, and conscious change etc. The median range hospital stay was 10 days (range: 0–41 days), with 40 patients discharged in stable condition and the remaining 38 patients deceased in the hospice ward only. Sedatives are widely used in the palliative care of terminally ill patients for treating insomnia, controlling delirium, and relieving terminal restlessness and agitation. While 39 patients required sedative medication for less than 30% of their hospital stay, the other 39 patients required sedatives for more than 30% or even greater than 70% of their stay.

Opioids are also common medication for patients in palliative care for controlling the pain and relieving dyspnea. The ideal strategy for pain control is to confine breakthrough pain three times a day while a single dose for breakthrough pain is about one-sixth of the daily dose of opioids. Therefore, increasing the use of opioids was viewed as over 50% increase from the daily dose before admission during the whole hospital stay. In this study, 47 patients had increasing demand of opioids compared to their previous prescription, while another 29 patients remained stable, and the other 2 patients decreased the need. Antipyretics are used to relieve infection or cancer-reduced fever; 60 patients were found with no or limited use (<30% of time) of antipyretics. At the time of admission, the physicians evaluated each patient’s KPS and prognostic tools. The KPS and PPS scales ranged between 70 and 10, with no patients scoring higher than 80 (see Table 1).

### 3.2. Description of Clinical Data

**Figure 1:**
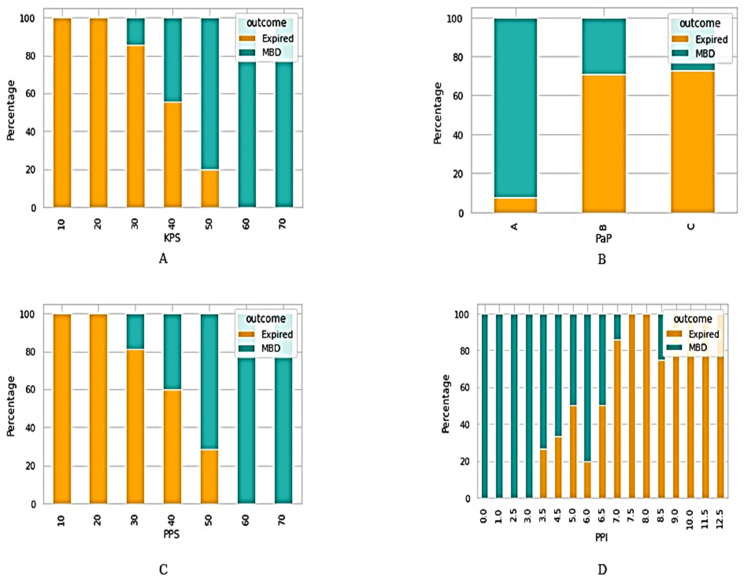
Classification of patients on the basis of prognostic tools. (**A**) KPS: Karnofsky Performance Status score-based classification; PaP: (**B**) Palliative Prognostic Score based classification; (**C**) PPS: Palliative Performance Scale based classification; (**D**) PPI: Palliative Prognostic Index based classification.

**Figure 2:**
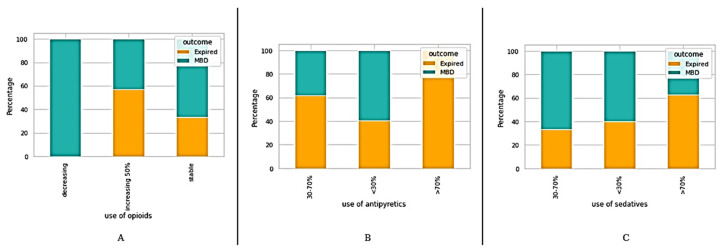
Classification of patients on the basis of medications—(**A**) Opioids (**B**) Antipyretics (**C**) Sedatives.

**Figure 3:**
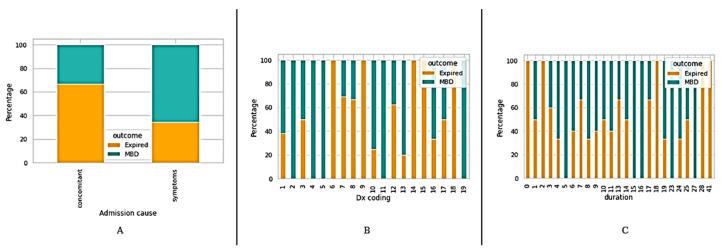
Classification of patients based on (**A**) admission cause, (**B**) diagnosis coding, (**C**) duration.

Patients were classified with regard to the survival outcomes (MBD and expired) on the basis of different clinical information, such as traditional prognostic tools including KPS, PaP, PPS, and PPI (see Figure 1). In addition, we also classified the patients based on their medications (see Figure 2); admission causes, diagnosis coding (Dx coding), and duration (length of stay) (see Figure 3).

### 3.3. Correlation of Clinical Information with Survival Outcomes

**Figure 4:**
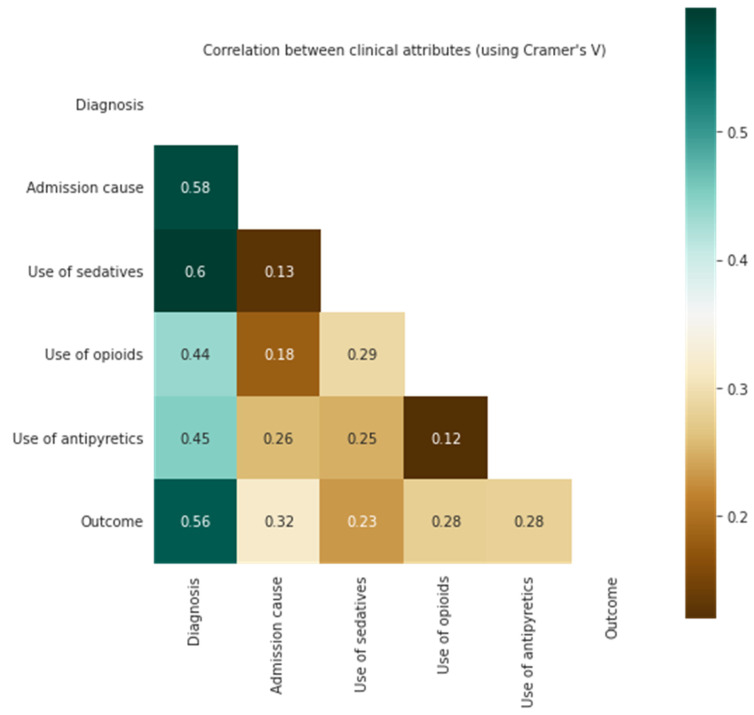
Correlation of clinical information with survival outcomes.

The evaluation of the patients’ age, sex, diagnosis, admission cause (comorbidities and symptoms), and the medication (sedatives, opioids, and antipyretics) were assessed using Crammer’s V correlation. The results indicated that patients with higher age and diagnostic scores were more correlated with the outcomes (see Figure 4); other factors, such as sex and admission cause, were also calculated; nevertheless, all of these factors, including the use of sedatives and the increased demand for opioids, relatively less correlated.

### 3.4. Description of Wearable Dataset

**Figure 5:**
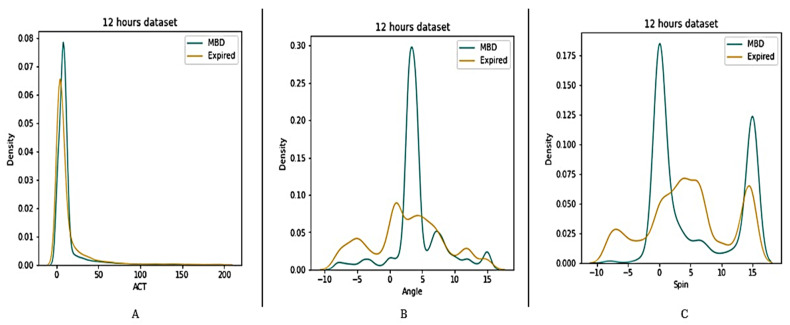
Description of wearable dataset at 12 h interval based on (**A**) ACT, (**B**) Angle (**C**) Spin.

**Figure 6:**
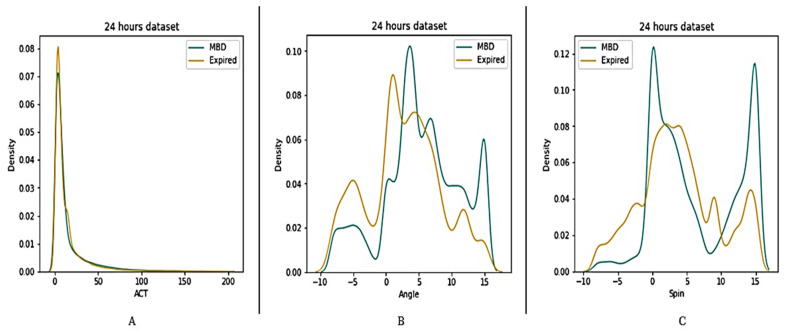
Description of wearable dataset at 24 h interval based on (**A**) ACT, (**B**) Angle (**C**) Spin.

**Figure 7:**
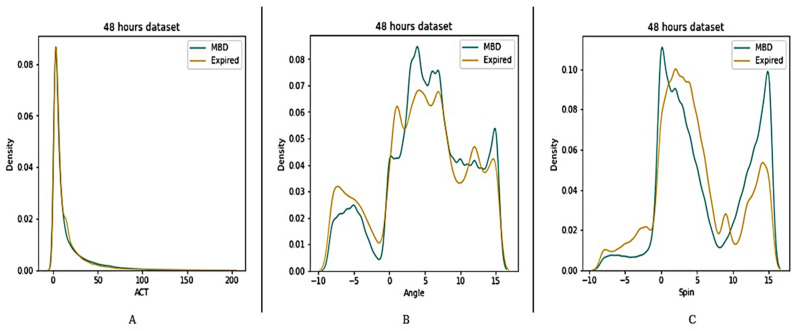
Description of wearable dataset at 48 h interval based on (**A**) ACT, (**B**) Angle (**C**) Spin.

**Figure 8:**
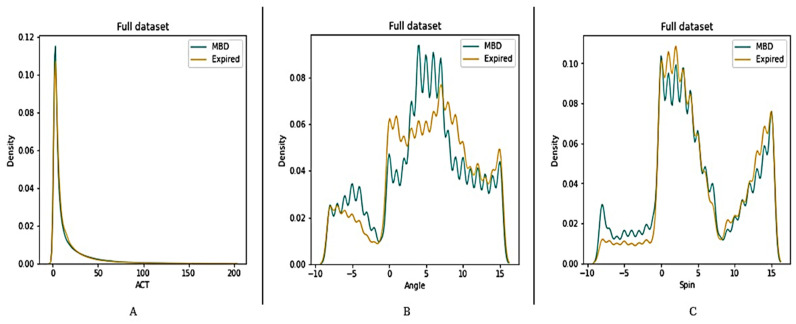
Description of wearable dataset at full dataset interval based on (**A**) ACT, (**B**) Angle (**C**) Spin.

Four datasets (12 h, 24 h, 48 h, and full dataset) were created in order to better understand the features of MBD and expired patients (see Figure 5, Figure 6, Figure 7 and Figure 8). The ACT value distributions for MBD and expired individuals were relatively similar, but angle and spin showed minor variances. In the first 12 h, both angle and spin revealed significant distribution variations between MBD and expired individuals (see Figure 5).

### 3.5. Significance of Cumulative Sensor Data to Differentiate between MBD and Expired

**Figure 9:**
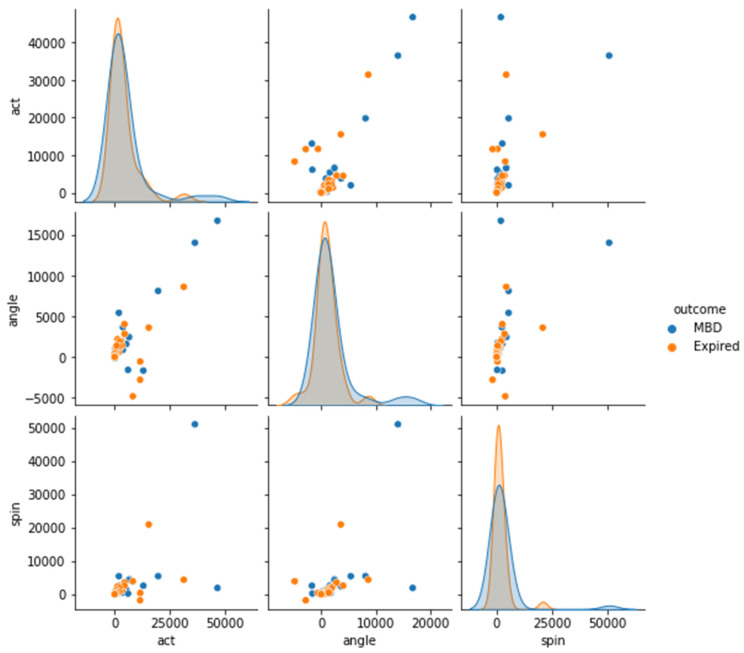
Differential representation between the ACT, angle, and spin at 12 h in order see the relationship between two variables (MBD and expire).

We differentiated the patients based on the MBD and expired using the pairplot to visualize the relationship of MBD and expired in reference to the ACT, angle, and spin values; and there is no difference found between outcomes (MBD or expired) in the 12 h dataset (Figure 9). The patients who survived had higher ACT, angle, and spin values as compared to those who expired. Although ACT, angle, and spin mean values of MBD, and expired patients have some differences for 12 h data, but their p values are >0.05 (Table 2). Thus, statistically there were no significant differences in ACT, angle, and spin values for MBD and expired patients in 12 h data (Figure 9).

**Figure 10:**
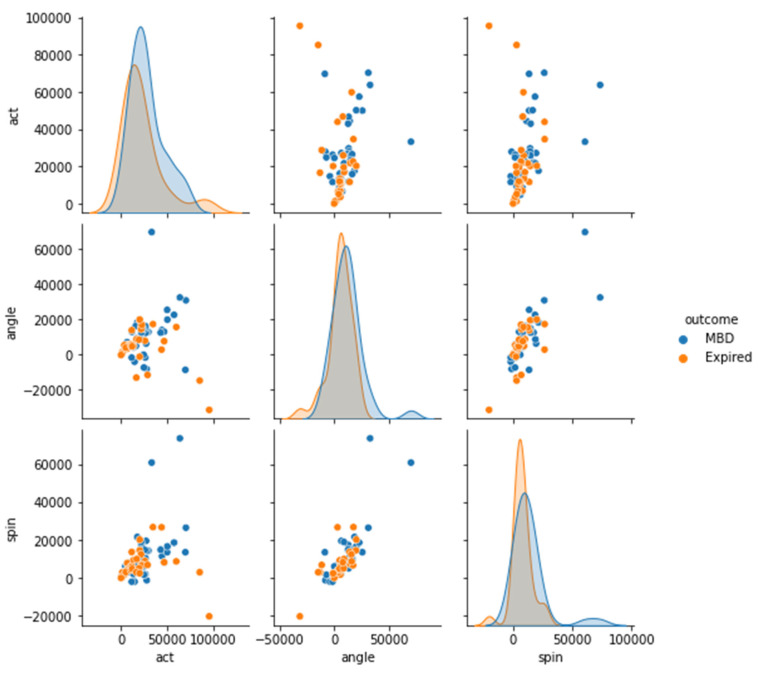
Differential representation between the ACT, angle, and spin at 24 h in order see the relationship between two variables (MBD and expire).

**Figure 11:**
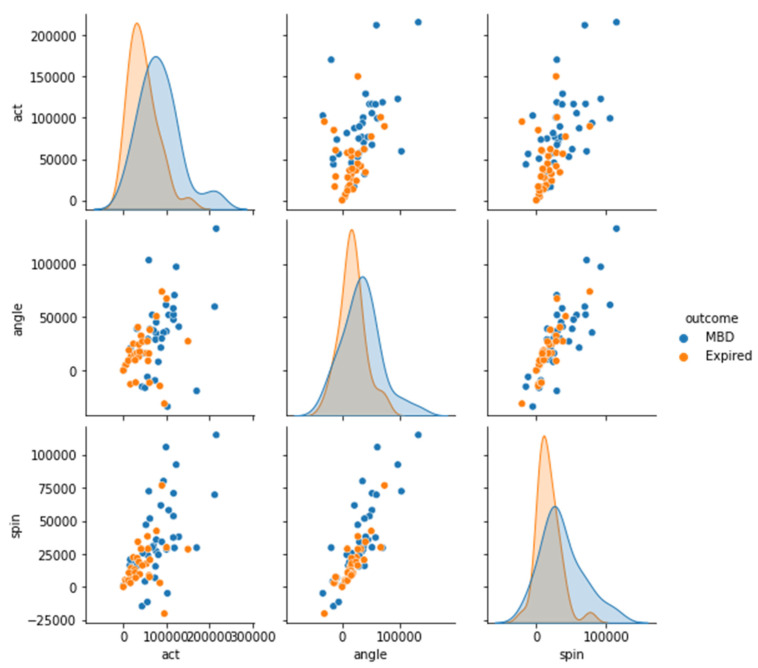
Differential representation between the ACT, angle, and spin at 48 h in order see the relationship between two variables (MBD and expire).

The relationship between MBD and expired in reference to the ACT, angle, and spin values in 24 h and 48 h dataset, has shown better difference in comparison with the 12 h dataset (Figure 10 and Figure 11). There is a significant statistical difference between the ACT, angle, and spin within the first 24 h, only angle p value is <0.05 (Table 3). Thus, statistically there are significant differences in total ACT, angle, and spin values for MBD and expired patients for 48 h data (Table 4).

## 4. Discussion

### 4.1. Mobile Technology in Palliative Care

The patients in our study were inevitably weak and needed care all the time (the majority of the patients had a KPS score lower than 50). Our results still suggested high adherence rate and data retrieval, thus demonstrating the success of implementing electronic activity monitors (EAMs) with Wearable Devices (WDs) for objective and continuous evaluation of PS. Several studies in the literature have already suggested feasibility of EAMS in cancer patients with adherence rate from 60–100% with various WDs for wrist, thigh, hip, or waist [21,30,31]. Earlier research tended to be descriptive and feasibility studies [30], while later publications discussed more about the correlation of physical activity (PA), quality of life (QOL) with questionnaires and EAMS [31]. With the advancements in technology, WDs were improved from only one-axial piezoelectric accelerometer to more modern features including: more dimensional electronics accelerometer with low-pass filter against external vibration and noise, detectors for electrocardiography or photoplethysmography for heart or pulse rate as well as oximeter for oxygen saturation and respiratory rate, more convenient wireless data transmission, larger battery and data storage, and more advanced algorithm to further monitor daily activity, evaluate QOL and interpret sleep duration [30]. The devices were also much less expensive and more reliable and popular in use, thus resulting in more research after 2016, as there were only less than five publications per year before 2015 but it increased rapidly from 14 papers in 2016 to 56 papers in 2020. With more innovative features, the recent investigation explored more diverse factors in daily life (including sleep quality, sedentary behavior and symptom burden), and lifestyle modifications such as moderate-to-vigorous physical activity (MVPA) and preventive care management [21,30].

Although with the increasing studies in recent years, the research focusing on WDs for the patients in palliative care was limited. To et al. (2019) first shared their experience in using commercial WDs for activity monitoring for the patients in palliative care; yet this brief report contained only 7 patients with no rigorous study design [32]. Pavic et al. (2020) demonstrated feasibility of continuous monitoring of cancer patients in palliative care with the WD as a bracelet and smartphone with apps; but this 30-patient study had 83.3% adherence rate and suggested a different heart-rate pattern for the patients needed emergency visits [33]. Meanwhile, another study required the patients to use both the bracelet was worn 53% and the smartphone was worn 85% of all study’s days [15]. In comparison, we simplified the patients’ use of the WDs to facilitate the trial. The patients were only required to wear the WD on the wrist all the time while the study nurse would help wirelessly collect the data, replace a new WD for low battery, and track the compliance of the use. In summary, EAMS with WD was feasible for objective and continuous evaluation of PS, especially for including patients in palliative care.

### 4.2. Need for Objective Prediction of Survival

The limitations of predicting survival tools in palliative care have been widely explored by many research, which have suggested various adjustments [34]. Although clinician predicted survival (CPS) was popularly advised in clinical settings, the accuracy of CPS was also under debate [34]. Stone et al. (2021) investigated the accuracy between CPS and four commonly used prognostic tools, including PaP score, PPS, PPI, and Feliu prognostic nomogram (FPN), demonstrating none of these prognostic tools was superior to CPS [35]. The study also revealed that the predicting tools were less sufficient in clinical use. The KPS and PPS were less distinguishing for score 30–50, though a trend inclined higher score with better outcome. PaP score and PPI suggested the survival probability yet there were alive patients in both worse risk groups and deceased patients in both promising risk groups. Although all predicting tools for survival are insufficiently accurate in clinical use, the study still found high correlation between predicting tools and outcome.

Stone et al. (2022) further explored the accuracy of CPS in a large clinical study with 1833 patients in palliative care, 431 physicians and 777 nurses [36]. This latest article suggested that both professional groups were better than the chance of in survival prediction; especially, the performance of clinician was “very good” at predicting imminent death, not surprisingly corresponding to common understanding. Even though CPS has been under justification for its importance, clinicians’ experience in palliative care and inter-observers’ bias are still inevitable [34]. The average years since qualified were 9 years for doctors and 19 years for nurses while the average years working in palliative care were 3 years for doctors and 6 years for nurses in the Prognosis in Palliative Care Study (PiPS) in the UK [35,36]. The PiPS compared different survival predictions between doctors and nurses for the same patients, suggesting doctors were more accurate than nurses. Yet the PiPS did not compare survival prediction between different doctors. Therefore, objective prediction beyond imminent death is needed.

Incorporating a dataset from WDs to predict survival is novel in exploring its use in healthcare. The Swiss study of using WDs and apps for the patients in palliative care in 2020 discovered a minor change of heart rate of the patients who needed emergency visits, suggesting WDs could warn the deteriorating condition in terminally ill patients. Tedoesco et al. (2021) first presented that a feature subset with limited factors including wearable dataset could suggest similar performance in predicting 2–7 year cancer-related mortality in older population compared to a full-featured dataset [37]. Another study suggested machine learning (ML) based models with wearable dataset could help in early detection of sepsis mortality in low-middle income countries (LMIC) [38]. Gresham et al. (2018) also used WDs to assess PS in advanced cancer patients, suggesting MVPA as each 1000 steps/day increase would help QOL and prolong survival (with hazard ratio: 0.48) [39]. Conclusively, the WDs has high potential in suggesting deterioration, morality, and prognosis for the patients in palliative care.

### 4.3. WD for Short-Term Survival

Our finding discovered more angle and spin movement for alive patients, while both alive and expired patients had similar wrist activity in 12, 24, and 48 h datasets. Although correlation between spin and outcome gradually decreased with longer time-sets, angle movement still had a strong association with the outcomes. Continuous wrist movement monitoring was challenging, and minor changes in angle and spin were considerably more difficult. However, WD as actigraphy assisted in resolving the issue. Although further investigation was required, our hypothesis for the difference was that angle and spin movement required more muscle power, implying that living patients still had superior PA in minor movements than expired patients. Better PA suggested better survival. This hypothesis corresponded to early detection of angle and spin difference between alive and expired patients; and it was also applicable to the poor differentiation of the patients with the KPS score 30-50. The KPS was grossly evaluated once at admission with less continuous and objective EAMS with WD while other prognostic tools also shared the same limitation. Moreover, medication, especially sedatives and opioids, was a concern to interfere with survival among clinicians though pathophysiology and pharmacologic mechanisms suggested little connection. The analysis confirmed frequent use of sedatives and increasing use of opioids had little correlation to the outcome; dying patients may have deteriorating symptoms but worsening symptoms did not definitely mean the patients were dying.

### 4.4. Risks of using Wearable Devices in Palliative Care

It must be understood that there are many ethical risks/practical aspects associated with the usage of wearable devices in palliative care settings. These risks must be considered before the deployment of the wearable devices for patient monitoring and data collection. As described by Low [40], some of these risks/challenges include but are not limited to: patients’ data protection, privacy, and security risks; digital literacy aspects for the elderly cancer patients; technology access and interrupted Wi-Fi issues in data syncing and capture; and level of comfort of patients for using wearable devices in the long term. Moreover, contrary to the studies providing potential benefits of the wearable devices, some studies in the literature have also questioned the accuracy of the data provided by wearable devices. Amin et al. [41] described some of the related studies and summarized few of those aspects that include: inaccuracy for the patients with slow and minimized movements; failure of accelerometers for fast movements; inaccurate assessment of step counts; requirement of frequent charging and syncing of data; and malfunction, damage, and loss of these wearable devices.

### 4.5. Study Limitations

Although limited by a small number of patients, single institute, and limited wearable dataset, this study still managed to demonstrate significant findings. Due to the COVID pandemic in the past 2 years, more patients in palliative care tended to expire at home. Moreover, human resource of the hospice and palliative care unit was also re-deployed; some physicians and nurses were assigned to other units for treating COVID patients or support fighting against the pandemic. This limited the study and decreased the recruitment. In addition, the recruitment only focused on cancer patients in palliative care, rather than other diseases and elderly patients, as the cancer patients often required more palliative care. Moreover, limited wearable dataset with only activity, angle, and spin metrics restricted further analysis. The wearable devices in this trial were lab-produced; thus, improvement was limited, and each improvement requires further validation in clinical use. Some commercial WDs, which are already validated in clinical settings, are often capable of detecting clinical features, while the accuracy, sensibility, and functionality would continue to improve over time. Further study using commercial WDs should be the trend, rather than developing a prototype. Furthermore, losing WD was an unexpected event, yet this experience would help future study designs to confirm data collection during the study. Owing to the nature of observational study, this study shared the inevitable biases including selection bias, information bias, confounding and internal validity. Rigorous prospective observational studies/randomized control trials with a larger number of participants or randomized control trials are recommended for the future investigations. 

## 5. Conclusions

The wristband actigraphy devices have great potential as prognosis tools in predicting the survival of the end-of-life patients in palliative care. Our study findings discovered more angle and spin movement for alive patients, while both alive and expired patients had similar wrist activity in 12, 24, and 48 h datasets. Although the correlation between spin and outcome gradually decreased with longer timesets, angle movement still had a strong association with the outcomes. The angle and spin movement required more muscle power implying living patients still had superior physical activity in minor movements than expired patients. The analysis also confirmed frequent use of sedatives and increasing use of opioids had little correlation to the outcome. Despite our study limitations, the results still showed high adherence rate and data retrieval, thus demonstrating the success of EAMs with WD for objective and continuous evaluation of performance status of the patients in palliative care. For the future study designs and investigations in this field, we recommend the utilization of commercial WDs, consideration of various biases, and recruitment of a large number of participants for better outcomes. On a side note, along with the potential uses and applications of wearable devices for the care of terminal patients, the related risks, e.g., patient privacy and cyber-security risks, along with the technical issues, e.g., connectivity and interoperability aspects, must be understood and addressed. Therefore, the judicious utilization of wearable devices is a sine qua non in critical settings such as palliative care for the safe, productive, and quality outcomes.

## Figures and Tables

**Table 1:** Characteristics of recruited patients (*p* < 0.05 is considered as significant *).

**Patients; *n* =**	78
**Age (years); mean (range)**	72.1 (39–92)
**Gender; *n* = (%)**	
Male	46 (58.97%)
Female	32 (41.03%)
**Primary Site of Cancer; *n* =**	
Bladder cancer	1
Brain cancer	2
Breast cancer	4 *
Cervical cancer	2
Cholangiocarcinoma	1
Colorectal cancer	19 *
Duodenal cancer	1
Endometrial cancer	1
Esophageal cancer	4
Gastric cancer	8
Hepatocellular carcinoma	3
Head and neck cancer	3
Lung cancer (non-small cell)	14
Small cell lung cancer	2
Ovarian cancer	2
Pancreatic cancer	2
Prostate cancer	9
Vaginal cancer	1
**Admission Cause; *n* = (%)**	
Concomitant diseases	34 (45.59%)
Cancer-related symptoms	44 (56.41%)
**Duration (days); median (range)**	10 (0–41)
**Outcome; *n* = (%)**	
Discharge	40 (51.28%)
Death	38 (48.72%)
**KPS score; *n* =**	
10	6
20	5
30	15
40	15
50	27
60	9
70	1
**PaP score group; *n* =**	
A	26
B	37
C	15
**PPS score; *n* =**	
10	1
20	7
30	17
40	16
50	23
60	11
70	3
**PPI score; *n* =**	
A	27
B	21
C	30
**Use of sedatives (time of study days); *n* =**	
<30%	39
30–70%	11
>70%	28
**Status of using opioids; *n* =**	
decreasing use	2
stable	29
increasing use	47
**Use of antipyretics (time of study days); *n* =**	
<30%	60
30–70%	14
>70%	4

KPS: Karnofsky Performance Status; PaP: Palliative Prognostic Score; PPS: Palliative Performance Scale; PPI: Palliative Prognostic Index.

**Table 2:** Statistical difference in ACT, angle, and spin values for MBD and expired in 12 h data.

Group Statistics						Independent Sample Test	
	Outcome	N	Mean	Std. Deviation	Std. Error Mean	t	*p*
ACT	MBD	37	4609.9086	9892.71842	1626.35289	0.497	0.621
	Expired	31	3578.8661	6493.51718	1166.27011		
angle	MBD	37	1779.49	3745.058	615.684	1.171	0.246
	Expired	31	885.90	2185.059	392.448		
spin	MBD	37	2466.19	8344.602	1371.844	0.569	0.571
	Expired	31	1539.81	3830.535	687.984		

ACT: Acceleration; MBD: Discharged.

**Table 3:** Statistical difference in ACT, angle, and spin values for MBD and expired in 24 h data (*p* < 0.05 is considered as significant *).

Group Statistics						Independent Sample Test	
	Outcome	N	Mean	Std. Deviation	Std. Error Mean	t	*p*
ACT	MBD	37	27,819.4646	18,034.62067	2964.87338	1.024	0.310
	Expired	31	22,762.5619	22,696.78910	4076.46366		
angle	MBD	37	11,304.95	14,008.048	2302.909	2.063	0.043 *
	Expired	31	4921.42	10,943.677	1965.542		
spin	MBD	37	12,946.70	14,883.926	2446.902	1.929	0.058
	Expired	31	7149.94	8318.599	1494.065		

**Table 4:** Statistical difference in ACT, angle, and spin values for MBD and expired in 48 h data (*p* < 0.05 is considered as significant *).

Group Statistics						Independent Sample Test	
	Outcome	N	Mean	Std. Deviation	Std. Error Mean	t	*p*
ACT	MBD	37	83,090.4759	48,290.05494	7938.83613	3.659	<0.001 *
	Expired	31	45,445.1471	33,616.47689	6037.69748		
angle	MBD	37	32,920.30	34,666.121	5699.075	2.197	0.032 *
	Expired	31	16,984.23	22,592.679	4057.765		
spin	MBD	37	36,702.27	30,719.356	5050.231	3.197	0.002 *
	Expired	31	16,919.39	16,983.133	3050.261		

## Data Availability

The raw data supporting the conclusions of this article will be made available by the authors, without undue reservation.
